# Tumor budding in gastric adenocarcinoma: prognostic value and association with clinicopathological markers

**DOI:** 10.1590/0102-67202025000048e1917

**Published:** 2025-12-19

**Authors:** Dhouha BACHA, Neirouz KAMMOUN, Bilel TROUDI, Monia ATTIA, Ahlem LAHMAR-BOUFAROUA, Sana BEN-SLAMA

**Affiliations:** 1 Mongi Slim Hospital, Pathology Department – Marsa, Tunis, Tunisia.; 2 Mongi Slim Hospital, Surgery Department – Marsa, Tunis, Tunisia.; 3 Mongi Slim Hospital, Radiology Department – Marsa, Tunis, Tunisia.

**Keywords:** Gastric Mucosa, Pathology, Gastric Neoplasms, Adenocarcinoma, Prognosis, Mucosa Gástrica, Patologia, Neoplasias Gástricas, Adenocarcinoma, Prognóstico

## Abstract

**Background:**

The analysis of tumor budding (TB) and its prognostic value in gastric adenocarcinoma (GA) has been the focus of several studies, with inconsistent results. This parameter is not included in gastric prognostic classifications or standardized pathological reports.

**Aims:**

To evaluate TB in GA and its prognostic significance through survival analysis, in addition to investigating the association between TB and clinicopathological markers that are considered prognostic factors for this type of cancer.

**Methods:**

This retrospective study covers a period of ten years, from January 2008 to December 2017. It included patients who underwent surgery for GA. TB evaluation followed the 2016 consensus guidelines for colorectal cancer, with three grades: Bd1 (0–4 buds), Bd2 (5–9 buds), and Bd3 (10 or more buds). Additionally, a two-grade classification system was employed, distinguishing between low-grade budding (fewer than 10 buds) and high-grade budding (10 or more buds).

**Results:**

TB was classified as low-grade in 69% of the cases and high-grade in 31%. High-grade TB was significantly correlated with perineural invasion (HR [hazard ratio]: 2.98, 95%CI [95% confidence interval] 1.04–8.53, p=0.004), stages III and IV (HR 4.04, 95%CI 1.27–12.83, p=0.01), and mortality (HR 3.65, 95%CI 1.24–10.74, p=0.02). It was an independent prognostic factor for recurrence-free survival (RFS) (p=0.005, p<0.05).

**Conclusions:**

We have demonstrated that TB prognostic and predictive value in GA is significant, particularly regarding patient survival.

## ARTICLE HIGHLIGHTS

Tumor budding (TB), a specific marker, is characterized by the presence of isolated cells or clusters containing fewer than five carcinoma cells at the invasive front of the tumor;

It is used as a prognosis marker in esophageal and colorectal disease, but not yet systematically used in gastric cancers;

A retrospective study including 68 patients who had undergone surgery for gastric adenocarcinoma was performed;

According to the study findings, overall survival, recurrence-free survival, and advanced tumor stages were significantly lower in patients with high-grade TB compared to those with low-grade TB;

We conclude that TB should be included in prognostic classifications and standardized reports of resected specimens of this cancer.

## Summary

**CENTRAL MESSAGE:**

In our series of 68 patients with gastric cancer, tumor budding was correlated with poor histopathological features and survival outcomes, supporting its role as a valuable prognostic factor.

**PERSPECTIVES:**

The evaluation of tumor budding in gastric cancer is a simple and cost-effective method. It should be incorporated into prognostic classifications and standardized pathology reports.

## VISUAL ABSTRACT

**Figure:**
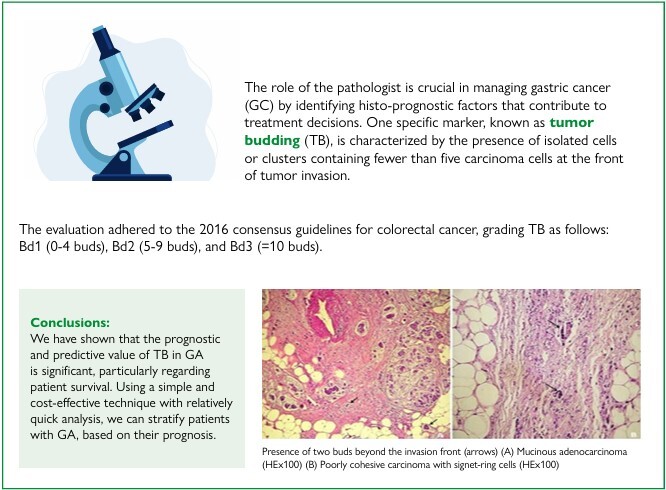

## RESUMO

**Racional:**

A análise do *tumor budding* (TB) e seu valor prognóstico no adenocarcinoma gástrico (AG) tem sido foco de diversos estudos, com resultados inconsistentes. Este parâmetro não é incluído nas classificações prognósticas gástricas nem em relatórios patológicos padronizados.

**Objetivos:**

O objetivo foi avaliar o TB no AG e sua significância prognóstica por meio de análise de sobrevida. Além disso, buscamos examinar a associação entre o TB e marcadores clínico-patológicos considerados fatores prognósticos para este tipo de câncer.

**Métodos:**

Este estudo retrospectivo abrangeu um período de dez anos, de janeiro de 2008 a dezembro de 2017, incluindo pacientes submetidos à cirurgia para AG. A avaliação do TB seguiu as diretrizes consensuais de 2016 para câncer colorretal, com três classificações: Bd1 (0-4 brotos), Bd2 (5-9 brotos) e Bd3 (10 ou mais brotos). Também utilizamos um sistema de classificação binário, diferenciando TB de baixo grau (menos de 10 brotos) e alto grau (10 ou mais brotos).

**Resultados:**

O TB foi classificado como de baixo grau em 69% dos casos e alto grau em 31%. O TB de alto grau apresentou correlação significativa com invasão perineural (HR 2,98, IC95% 1,04–8,53, p=0,004), estádios III e IV (HR 4,04, IC95% 1,27–12,83, p=0,01) e mortalidade (HR 3,65, IC95% 1,24–10,74, p=0,02). Foi identificado como um fator prognóstico independente para a sobrevida livre de recorrência (SLR) (p=0,005, p<0.05).

**Conclusões:**

Demonstramos que o valor prognóstico e preditivo do TB no AG é significativo, especialmente em relação à sobrevida dos pacientes.

## INTRODUCTION

 Gastric cancer (GC) is the fifth most common cancer worldwide and the fourth leading cause of cancer-related deaths^9^. In Tunisia, it has a five-year prevalence estimated at 7 cases per 100,000 inhabitants and a cumulative risk of mortality estimated at 0.34^6,8^. Despite advancements in diagnostic tools and treatment options, GC is frequently diagnosed at advanced stages, resulting in a poor prognosis and a five-year survival rate of less than 30%^20^. 

 The role of the pathologist is crucial in managing GC by identifying histo-prognostic factors that contribute to treatment decisions. Among these factors, markers of epithelial-mesenchymal transition, an important process in tumor progression, represent a new challenge for pathologists. One specific marker, known as tumor budding (TB), is characterized by the presence of isolated cells or clusters containing fewer than five carcinoma cells at the invasive front of the tumor. The analysis of TB and its prognostic value has been the subject of several studies, focusing on various cancers, particularly esophageal and colorectal cancers^17,22,27,26^. 

 In most of these studies, TB was associated with a poor prognosis, significantly reducing both overall and relapse-free survival. However, the results are inconsistent in GC, and this parameter is not included in the prognostic classifications, nor is it present in standardized pathological reports^10,14^. 

 In this study, we aimed to evaluate TB in gastric adenocarcinoma (GA) and to assess its prognostic value through survival analysis. 

 The objective is to explore the associations between TB and commonly recognized clinicopathological markers that are considered prognostic factors for this type of cancer. 

## METHODS

 This retrospective study comprised a period of ten years, from January 2008 to December 2017. All patients who underwent surgery for GA at the Mongi Slim Hospital, in Tunisia, were included. The focus of the study lies on various clinicopathological factors, endoscopic features, tumor staging, and treatment details. Patients whose tumor sample slides were lost or whose paraffin blocks were depleted were excluded from the study. The pathological characteristics of the tumors were assessed using the Lauren classification and the WHO classifications from 2010 and 2019^15,16^. The pathological stages (pTNM) were determined according to the 2017 criteria set by the Union for International Cancer Control (UICC) and the American Joint Committee on Cancer (AJCC)^11,15^. Additionally, the presence of perineural invasion, vascular emboli, and the patient’s response to chemotherapy, evaluated through the Mandard Tumor Regression Grade (TRG), were documented^19^. 

### Tumor budding assessment

 For TB assessment, histological slides from patients with GA were reexamined by a single pathologist (DB), focusing on the invasive fronts of the tumors. These slides were stained with hematoxylin and eosin (HE). 

 TB was defined as the presence of isolated cells or small clusters of fewer than five cells at the invasive front of the tumor, as commonly used in the scientific literature^30^. The evaluation followed the 2016 consensus guidelines for colorectal cancer, grading TB as follows: Bd1 (0–4 buds), Bd2 (5–9 buds), and Bd3 (≥10 buds). Furthermore, a two-grade classification was employed, categorizing budding as low-grade (<10 buds) and high-grade (≥10 buds)^18^. 

### Statistical analysis

 For the statistical analysis, the Statistical Package for the Social Sciences (SPSS) statistical software was used. The confidence interval was set at 95% (95%CI). Continuous quantitative variables were represented by mean and standard deviation, while qualitative variables were expressed as percentages. To compare means, the Student’s t-test was employed. Associations between TB and specific clinicopathological factors were evaluated using Pearson’s χ² test. A correction was applied using Fisher’s exact test when the sample size was less than five. To identify risk factors that were independently associated with TB, a multivariate analysis using Cox regression, following a stepwise descending approach, was carried out. This analysis included all factors that had a significance level of p< 0.2 in the univariate analysis. For follow-up and survival analysis, survival curves for overall survival (OS) and recurrence-free survival (RFS) were created using the Kaplan-Meier method, and these curves were compared using the log-rank test. The study was approved by the Ethics Research Committee of the Institution (number 24/082). 

## RESULTS

 Our research involved 68 patients who had undergone surgery for GA. The median age of the patients was 61.34 years (±13.58), with the highest incidence occurring between the ages of 61 and 70 years. The cohort consisted of 49 men (72%) and 19 women (28%), resulting in a male-to-female ratio of 2.57. The postoperative course was uneventful for 60 patients (88%), while eight cases (12%) required revisional surgery. 

 The clinicopathological features and patient management details are summarized in Table 1. The mean tumor size was 58.43±24.20 mm, with sizes ranging from 10 to 130 mm. In terms of TNM staging, 31 patients (45%) were classified as pT3, and 22 patients (32%), as pT4. Furthermore, 42 patients had lymph node metastasis, and 11 patients (16%) presented with synchronous distant metastasis. 

**Table 1:** Clinicopathological features of the study patients.

			n	%
Symptoms				
	Epigastric pain		57	84
	Hemorrhage		14	21
	Dysphagia		12	18
	Chronic anemia		17	25
	Nausea and vomiting		23	34
	Deterioration of general condition		37	54
Physical examination				
	Epigastric tenderness		33	49
	Palpable mass		3	4
	Unremarkable		32	47
Tumor location				
	Antrum		22	32
	Subcardial		13	19
	Small and large curvature		24	35
	Pangastric		7	10
Endoscopic appearance				
	Mass		4	6
	Ulcerative tumor		2	3
	Infiltrative ulcerative tumor		60	88
	Diffuse infiltrative tumor		2	3
Type of surgery				
	Total gastrectomy		45	66
	Subtotal gastrectomy		23	34
Lymph node dissection				
	D1.5		51	75
	D2		17	25
Surgical resection				
	R0		60	88
	R1		6	9
	R2		2	3
Perioperative CT (FLOT)			24	35
Adjuvant treatment (no neoadjuvant treatment)				
	CT		5	7
	Chemoradiotherapy		1	2
Tumor subtypes				
	Lauren’s classification			
		Intestinal	32	47
		Diffuse	30	44
		Mixed	6	9
	WHO 2019 classification			
		Tubular	27	40
		Papillary	4	6
		Mucinous	6	9
		Poorly cohesive carcinoma	25	36
		Mixed tumors	6	9
Tumor differentiation				
	WHO 2010			
		Well-differentiated	30	44
		Moderately-differentiated	13	19
		Poorly-differentiated	25	37
	WHO 2019			
		Low-grade	43	63
		High-grade	25	37
	Stages			
		IA	4	6
		IB	8	12
		IIA	10	15
		IIB	8	12
		IIIA	7	10
		IIIB	7	10
		IIIC	11	16
Vascular embolism			30	44
Follow-up				
	No complication		60	88
	Revisional surgery		8	12
	Local recurrence		12	18
	Metastatic recurrence		15	22
	Death		32	47

WHO: World Health Organization; R: resection; CT: chemotherapy; FLOT: Fluorouracil (5-FU), Leucovorin (folinic acid), Oxaliplatin, and Docetaxel.

**Figure 1:**
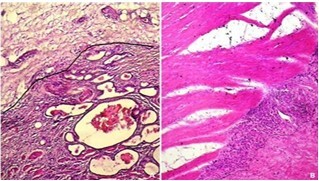
Well-differentiated and infiltrating tubular adenocarcinoma. **(A)** Poorly-cohesive carcinoma (HEx200); **(B)** Absence of tumor budding beyond the invasive front (lines) (HEx200).

**Figure 2:**
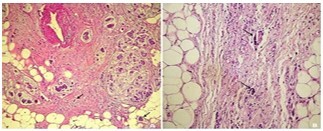
Presence of two buds beyond the invasive front (arrows). **(A)** Mucinous adenocarcinoma (HEx100); **(B)** Poorlycohesive carcinoma with signet-ring cells (HEx100).

 The TB was assessed as follows: Bd1 in 35 patients (51%) (Figures 1 and 2), Bd2 in 12 patients (18%), and Bd3 in 21 patients (31%). 

 For the two-grade classification, tumors were classified as low-grade in 69% of cases and high-grade in 31% of cases. Local recurrence was observed in 12 patients (18%) within an average of 17.17±14.87 months, while metastatic recurrences occurred in 15 patients (22%) within an average of 21.93±12.2 months. In our study, 32 patients (47%) died during the follow-up period, with a time frame ranging from 0 to 48 months post-surgery. 

### Association between tumor budding and survival

**Figure 3:**
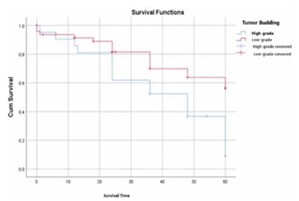
Overall survival curves depending on tumor budding in the study series. Cum: Kaplan-Meier curve for overall survival.

 The average OS for patients with high-grade TB was significantly lower than that of patients with low-grade TB (29.19 months vs 47.87 months; p=0.007, p<0.05) (Figure 3). 

**Figure 4:**
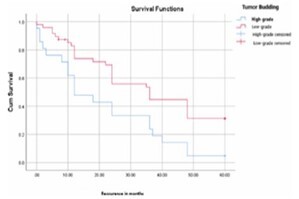
Recurrence-free survival curves depending on tumor budding in the study series. Cum: Kaplan-Meier curve for overall survival.

 Similarly, the mean RFS for patients with high-grade TB was also lower than that for patients with low-grade TB, showing a significant difference (21.04 months vs. 35.42 months; p=0.004, p<0.05) (Figure 4). 

 According to the multivariate analysis, high-grade TB was an independent prognostic factor for RFS (p=0.005, p<0.05). 

### Association between tumor budding and clinicopathological features

 The results of our univariate analysis are summarized in Table 2. In our study, we found a correlation between high-grade TB and several factors: poorly-differentiated tumors (p=0.035, p<0.05), vascular invasion (p=0.06, p>0.05), perineural invasion (p=0.038, p<0.05), deep parietal infiltration (pT3–pT4) (p=0.003, p<0.05), the occurrence of metastatic recurrence (p=0.033, p<0.05), nodal staging pN3 (p=0.005, p<0.05), synchronous distal metastasis (p=0.014, p<0.05), and stages III and IV (p=0.014, p<0.05). Additionally, high-grade TB was associated with metastatic recurrence (p=0.0033, p<0.05) and mortality (p=0.016, p<0.05). In the multivariate analysis, high-grade TB was significantly correlated with perineural invasion (odds ratio [OR] 2.98, 95%CI 1.04–8.53, p=0.004, p<0.05), advanced tumor stages (III–IV) (OR 4.04, 95%CI 1.27–12.83, p=0.01, p<0.05), and mortality (OR 3.65, 95%CI 1.24–10.74, p=0.02, p<0.05), as shown in Table 3. 

**Table 2:** Univariate analysis to identify the association of tumor budding with clinicopathological parameters in the study patients.

Prognosis factors		Low-grade TB	High-grade TB	p-value
Age (years)				
	<60	18	29	0.47
	≥60	10	11	
Sex				
	Men	33	16	0.17
	Women	14	5	
Size				
	<58 mm	14	8	0.17
	≥58 mm	33	13	
Poorly-differentiated tumor		11	14	0.035
Vascular invasion		15	15	0.006
Perineural invasion		15	13	0.038
Parietal infiltration (pT3/pT4)		32	21	0.003
Lymph node stage pN3		8	11	0.005
Synchronous distant metastasis		4	7	0.014
Stage III and IV tumour		21	17	0.014
Local recurrence		6	6	0.11
Metastatic recurrence		7	8	0.033
Death		17	15	0.016

TB: tumor budding.

**Table 3:** Multivariate logistic analysis to identify the association of tumor budding with clinicopathological parameters in the study patients.

Variables	HR	95%CI	p-value
Poorly-differentiated tumor	3.04	1.05–8.76	0.93
Vascular invasion	4.42	1.49–13.15	0.26
Perineural invasion	2.98	1.04–8.53	0.004
Stages III and IV tumor	4.04	1.27–12.83	0.01
Metastatic recurrence	3.51	1.06–11.58	0.13
Death	3.65	1.24–10.74	0.02

HR: hazard ratio; 95%CI: 95% confidence interval.

## DISCUSSION

 According to our findings, OS and RFS were significantly lower in patients with high-grade TB compared to those with low-grade TB, with p-values of 0.007 and 0.004, respectively. High-grade TB was identified as an independent prognostic factor for RFS (p=0.005, p<0.05). Furthermore, high-grade TB were independently associated with poor histopathological factors, including the presence of perineural invasion (p=0.004, p<0.05), advanced tumor stages (III–IV) (p=0.01, p<0.05), and increased mortality (p=0.025, p<0.05). 

 These results led us to investigate the impact of TB in GA, drawing on existing literature. 

 The presence of TB has become a significant factor in predicting the progression of colorectal cancer (CRC), especially in stage II. Patients in this stage could benefit from new prognostic factors that help classify them as either low or high risk for recurrence, which has therapeutic implications^27^. 

 In 2016, the UICC recommended including TB as a criterion for adjuvant treatment^2,33^. Since then, authors of several studies have sought to demonstrate the prognostic value of TB in other types of cancer, including GA^31^. It has been the focus of various studies. The findings are summarized in Table 4. 

**Table 4:** Prognosis value of tumour budding in gastric adenocarcinoma according to the main literature review.

Study	Number of patients	Factors correlated to high-grade TB	p-value
Ulase et al.^31^	456 patients	Sex (men)	0.002
		RFS in high-grade TB<low-grade TB	0.001
		Advanced parietal invasion	<0.001
		Advanced pN stage	<0.001
		Synchronous metastasis	0.001
		Lymph node metastasis	<0.001
		Perineural invasion	<0.001
		MSS status	<0.001
		Lower OS	<0.001
Du et al.^4^	621 patients with early gastric carcinoma	Lymph node metastasis	<0.01
Brown et al.^1^	356 patients	Lower OS	0.0001
		Poorly-differentiated tumor	0.0001
		Higher pT tumor stage	0.0001
		pN3 lymph node stage	0.0001
		Incomplete tumor resection	0.0001
Tanaka et al.^29^	320 patients	Tumor size	<0.01
		pT4 tumor stage	<0.01
		Moderately-differentiated tumor	<0.01
		Vascular invasion	<0.01
		pN+ lymph node stage	<0.01
		Synchronous metastasis	<0.01
Olsen et al.^24^	104 patients	Poorly-differentiated tumor	0.002
		Vascular invasion	<0.001
		Perineural invasion	0.002
		Higher pT tumor stage	0.001
		Higher pN stage	0.001
		Higher grade	0.002
		Recurrence	0.007
Che et al.^2^	296 patients	Lower OS	<0.001
		Poorly-differentiated tumor	<0.001
		pT(3–4) tumor stage	<0.001
		pN+ lymph node stage	<0.001
		Synchronous metastasis	0.005
		Higher tumor stage	<0.001
Kemi et al.^12^	583 patients	Lower OS	<0.001
		Younger age (64 vs 70)	<0.001
		Tumor stage (pT3–4)	<0.001
Dao et al.^3^	109 patients	Vascular invasion	0.003
		Perineural invasion	<0.001
		Recurrence	<0.001
		pN+ lymph node stage	<0.001
		Death	<0.001
		Lower OS	<0.001
		Lower DFS	<0.001
El Yaagoubi et al.^5^	83 patients	Age <68 years	0.02
		Incomplete tumor resection	0.03
		Vascular invasion	0.05
		Perineural invasion	0.04
		pN+ lymph node stage	0.04
		Advanced stage	0.02
		OS	0.04
		Recurrence	0.01
		Lower OS	0.04
		Lower RFS	0.01
Zhang et al.^32^	147 patients	Larger tumor size	0.003
		pN+ lymph node stage	<0.001
		Higher pT tumor stage	<0.001
Qi et al.^26^	153 patients	Age >60 years	<0.001
		Women	0.028
		pN+ lymph node stage	0.003
		Lymph node metastasis	0.039
		Worse OS	<0.001
Sun et al.^28^	122 patients	Poorly-differentiated	<0.0001
		Lymph node metastasis	<0.0001
		Higher tumor stage	0.007
Kucuk et al.^14^	43 patients	pN3 lymph node stage	0.001
		Poorly-differentiated tumor	0.001
Pun et al.^25^	76 patients	Higher pT tumor stage	<0.001
		Higher pN lymph node stage	0.004
		Higher grade	0.01
		Vascular invasion	<0.001
		Perineural invasion	0.002
Jesinghaus et al.^11^	176 patients	Lower OS	<0.001
		Advanced pT tumor stage	<0.001
		Advanced pN lymph node stage	0.045
		pM metastatic stage	0.050
		Higher tumor grade	0.005
		Incomplete resection R1	0.003

TB: tumor budding; RFS: recurrence-free survival; MSS: microsatellite stable; OS: overall survival; DFS: disease-free survival; pT: pathological primary tumor stage; pN: pathological nodal staging; pM: pathological distant metastasis.

 Guo et al., in a meta-analysis, demonstrated the impact of high-grade TB on survival. This analysis involved seven stud ies with a total of 2,178 patients. The authors showed that high-grade TB predicted a poor 5-year OS with OR of 1.79 (95%CI 1.53–2.05, p<0.01) for patients with GA^27^. According to the combined OR values, high-grade tumor burden was significantly associated with several factors: tumor stage (OR 6.63, 95%CI 4.01–10.98, p<0.01), tumor differentiation (OR 3.74, 95%CI 2.68–5.22, p<0.01), lymphovascular invasion (OR 7.85, 95%CI 5.04–12.21, p<0.01), and lymph node metastasis (OR 5.75, 95%CI 3.20–10.32, p<0.01)^27^. 

 Furthermore, Ulase et al., in a study including 456 patients, verified that those with high-grade TB experienced significantly reduced 5-year OS compared to those with low-grade TB (p<0.001)^31^. Similar findings were reported in another study by Du et al., involving 621 patients (p<0.001)^4^. 

 Authors of several studies have identified an association between high-grade TB and poor tumor differentiation^1,3,13,25,28-30^. According to other findings, there are correlations with the presence of vascular and perineural invasion^5,12,23,24,29,31^, while some researchers reported associations with older age^25^ and larger tumor size^28,32^. However, these latter findings were not observed in our study. 

 Variations in the literature results are primarily attributed to the significant differences in methods used to assess and quantify TB. Unlike CRC, there is currently no standardized method for evaluating this parameter in GA^4,23,25,30^. Several evaluation systems have been proposed, including one by Hase et al., which uses a binary classification based on the predominant morphological characteristics: either absent/minimal budding or moderate/marked budding^4^. Another system, developed by Nakamura et al., classifies TB into four scores based on the surface of the tumor’s invasive front^22^. In addition, quantitative systems, such as the one proposed by Ueno et al., focus on counting tumor buds in "hot spot" areas identified through low-magnification HE slides^30^. In our series, we employed the method validated in CRC, which distinguishes three grades based on the number of buds present. 

 It is essential to consider the reproducibility of these methods, as significant variability has been demonstrated among different evaluators, even when using the same technique^33^. 

 An immunohistochemistry (IHC) study using a pan-Cytokeratin antibody and image analysis (IA) technology can effectively identify carcinoma cells at the invasive front and aid in evaluating TB^25^. 

 However, it is worth noting that IHC involves additional costs in terms of both money and time. Assessing TB in GA can be particularly challenging in the presence of significant post-therapeutic fibrosis or when the distribution of this fibrosis is heterogeneous^25^. In such situations, distinguishing the invasive front between tumor tissue and adjacent healthy tissue from the junction of tumor remnants and post-therapeutic fibrosis may be difficult. In all cases, it is crucial to perform extensive sampling of the tumor along with adjacent tissue to analyze as many invasive-front areas as possible. 

 Our study has several limitations. It is a retrospective analysis based on available histological samples. During our investigation, we conducted a "prospective" re-evaluation of tumor slides to assess TB, as this parameter was not included in the routine histological reports. We chose not to use additional techniques, such as special staining, IHC, or IA. Instead, we adhered to the consensus for evaluating TB in CRC, which solely relies on histological examination of HE-stained slides. 

## CONCLUSIONS

 We have shown that the prognostic and predictive value of TB in GA is significant, particularly regarding patient survival. Using a simple and cost-effective technique with relatively quick analysis, we can stratify patients with GA based on their prognosis. To further validate this parameter, larger prospective studies are needed to explore its potential therapeutic implications for increasingly personalized treatment approaches in GA. With these findings, we highlight the need for a standardized method to assess TB in GA, allowing for its inclusion in prognostic classifications and standardized reports of resected specimens of this cancer. 

## Data Availability

The datasets generated and/or analyzed during the current study are available from the corresponding author upon reasonable request.
